# Optimized fabrication of a Y-doped Ti/TiO_2_ macroporous membrane electrode and its application in the electrosynthesis of succinic acid

**DOI:** 10.1039/d5ra03030g

**Published:** 2025-06-23

**Authors:** Shaojie Hong, Fanhua Yu, Bin Guo, Xiangqian Ren, Xingfu Zhou

**Affiliations:** a State Key Laboratory of Materials-Oriented Chemical Engineering, College of Chemical Engineering, Nanjing Tech University Nanjing 210009 China Zhouxf@njtech.edu.cn +86-25-83172270 +86-25-83172270

## Abstract

In recent years, “green synthesis” technology has emerged at the research forefront of the chemical industry as an environmentally friendly approach. As a new and effective chemical synthesis method, organic electrochemical synthesis technology has attracted increasing attention. In this paper, the sol–gel method is used to fabricate a Y-doped Ti/TiO_2_ electrode. By adding different concentrations of Y ions, the macropore morphology of the film becomes more obvious, and substrate cracks are improved to a certain extent. EIS tests show that a 0.006-Y electrode exhibits lower charge-transfer resistance. Furthermore, linear voltammetry (LSV) analysis showed that the hydrogen evolution potential of the Y-doped Ti/TiO_2_ film electrode was improved. At the optimal Y/Ti molar ratio of 0.006, hydrogen evolution potential reached −1.22 V, showing a −0.19 V shift compared with the undoped electrode, and the hydrogen evolution side reaction was effectively inhibited. Cyclic voltammetry (CV) tests show that the reduction peak current density in maleic acid solution is as high as 210 mA cm^−2^, which is 1.7 times that of the undoped electrode, indicating that the addition of Y at trace concentrations improves the electrocatalytic reduction performance of the electrode. Considering that the cathode can reduce maleic acid at lower potential and has higher catalytic activity, cathode potential is controlled within the range of −0.6–1.2 V for the electrosynthesis of succinic acid. When the optimized reaction temperature is 50 °C, the electrosynthesis yield for succinic acid reaches 91%, and the current efficiency reaches 96.3%.

## Abstract 2

Y-doped Ti/TiO_2_ electrodes were prepared for succinic acid electrosynthesis. Y doping enhanced electrocatalytic reduction, achieving 91% yield and 96.3% current efficiency at 50 °C.

## Graphical abstract

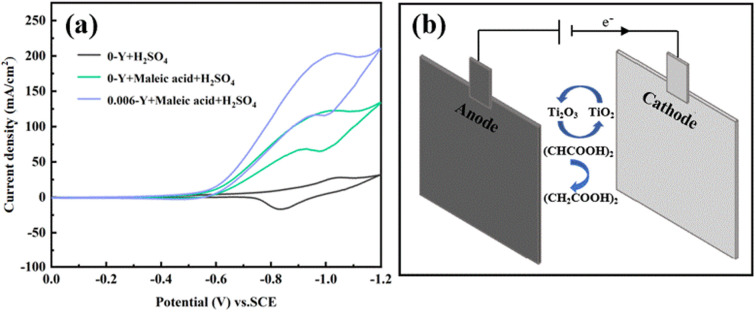

## 1. Introduction

Owing to its advantages of low cost, nontoxicity, high conductivity, high chemical stability, and high thermal stability, nano-titanium dioxide has been widely studied in the field of electrochemistry.^1–4^ In 1984, Beck *et al.*^5^ found that a TiO_2_ electrode could be used as an excellent REDOX carrier for the heterogeneous electrocatalysis of nitro compounds, showing high catalytic activity and a current efficiency of up to 70%, which made TiO_2_ widely attractive in the field of electrochemistry. In recent years, TiO_2_ electrodes have been used as electrocatalysts for the reduction of organic matter such as maleic acid,^6,7^ acetic acid,^8^ and nitrophenol.^9^ Ahmadi *et al.*^10^ demonstrated that TiO_2_ nanotube arrays showed excellent catalytic activity and high stability in the electrocatalytic reduction of nitrophenol (4-NP) to *p*-aminophenol (4-AP). However, the low conductivity of TiO_2_ is the main reason leading to its poor electrocatalytic activity, which limits the application of TiO_2_ in the field of electrocatalysis to a certain extent.

To overcome the inherent defects of the low conductivity of TiO_2_, people have extensively studied the engineering of TiO_2_ structures through the generation of oxygen vacancies,^2,11^ element doping,^12–14^ the formation of titanium suboxide and carbon composites,^15^ and other methods. These methods are aimed at improving the electronic conductivity of TiO_2_ to ensure charge transfer efficiency in electrochemical catalytic reactions. In recent years, element doping has been proven to be significantly effective in promoting reduction current and inhibiting electrode passivation.^16^ Normally, doping atoms can be introduced into the lattice of titanium dioxide and exist as oxides. Xu *et al.*^17^ prepared Ti/TiO_2_–ZrO_2_ membrane electrodes with high activity by sol–gel method, and the yield of butyric acid reached 96%. Wang *et al.*^18^ reported on the preparation of a Ce Ce-doped nano-TiO_2_ film electrode by the sol–gel method, which was used to study the electroreduction of maleic acid (MA) by a Ce nano-TiO_2_ film electrode in H_2_SO_4_ solution. The results showed that when the optimal molar ratio of Ce : Ti was 0.003 : 1, the peak reduction current was 4.5 times that of the undoped nano-TiO_2_ electrode, and the current efficiency of reducing succinate was as high as 91%. However, yttrium (Y) has been rarely used as the target doping element to promote the performance of the TiO_2_ film electrode electrocatalytic reduction.

In this study, by using yttrium trichloride (YCl_3_) as the preferred additive, we introduced a precursor solution containing the Ti source. After annealing and calcining the Ti base combined with the pulling method, we successfully prepared Ti/TiO_2_–Y_2_O_3_ electrodes with different Y doping concentrations. Further study confirmed that the doping of trace Y elements can effectively increase the hydrogen evolution potential of the TiO_2_ film electrode and increase the peak current of the cathode reduction. Subsequently, we discussed the electrocatalytic reduction mechanism of maleic acid to succinic acid by the Y-doped Ti/TiO_2_ film electrode, and the electrosynthesis of succinic acid at a controlled cathode potential, achieving a current efficiency of 96.3%. This work also confirmed that the optimization of doping elements is a reliable approach toward improving the electrocatalytic performance and cooperative stability of electrodes, and further promoted the development of Ti/TiO_2_ membrane electrodes in organic synthesis.

## 2. Experimental section

### 2.1 Reagents and materials

Tetrabutyl titanate (C_16_H_36_O_4_Ti), yttrium trichloride (YCl_3_), and maleic acid (C_4_H_4_O_4_) were purchased from Aladdin. All other reagents were purchased from Shanghai Maclin Biochemical Technology Co., Ltd.

### 2.2 Electrode preparation

First, the titanium plate (50 mm × 40 mm × 0.5 mm) was immersed in an alkali solution (10 wt% NaOH) with a steady temperature of 95 °C for 30 minutes to remove the grease on the surface, followed by a rinse process with deionized water. Then, the substrates were heated in a 10 wt% oxalic acid solution at 98 °C for 2 h. When a gray pitted surface was observed, the titanium plate was cleaned in water with ultrasonication for 0.5 h.

The TiO_2_ precursor solution was labeled as solution 1. Anhydrous ethanol was used as the solvent, tetra butyl titanate (0.45 M) was added, and yttrium trichloride (Y : Ti molar ratio *x* : 1, *x* = 0,0.002, 0.004, 0.006, 0.008) was successively dissolved in an anhydrous ethanol solution containing the titanium source. Solution 2 was then prepared as follows. Measured volumes of 30 mL anhydrous ethanol, 2.5 mL acetic acid (as the catalyst, controls the hydrolysis rate, inhibits the formation of precipitation), and 6 mL polyethylene glycol (as a stabilizer, prevents agglomeration) were mixed and thoroughly stirred. Finally, solution 1 was placed onto the magnetic agitator, the rotational speed was adjusted, and solution 2 was added into solution 1 dropwise to obtain a stable, uniform, clear and transparent light yellow solution. After the dropwise addition was finished, the mixture was stirred continuously for 3 hours before use and left to rest. After aging, the solution turned into a gel.

**Fig. 1:**
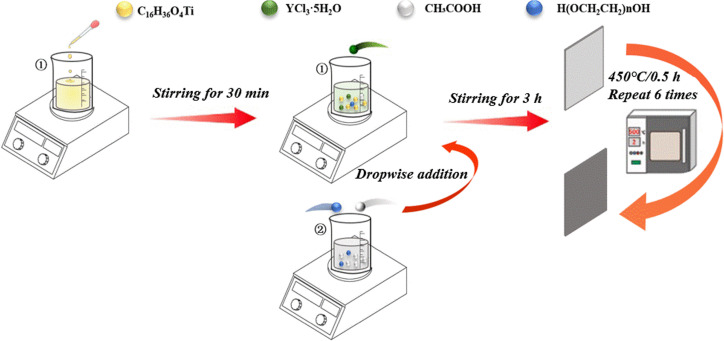
The preparation process of the Ti/TiO_2_ cathode.

The sol–gel was coated onto the etched Ti substrate by the lifting method, dried for 10 min in the oven at 120 °C, and then calcined for 30 min in the Muffle furnace at 450 °C. The process was repeated 6 times; that is, Ti/TiO_2_–Y_2_O_3_ electrodes with different doping ratios of Y elements were prepared. The prepared electrodes were recorded as 0-Y, 0.002-Y, 0.004-Y, 0.006-Y, and 0.008-Y electrodes based on the different doping ratios of Y. As shown in Fig. 1, it is a schematic diagram of the steps of this method.

### 2.3 Physicochemical characterization

A Hitachi S-4800 scanning electron microscope (SEM) model with a magnification of 9.7 mm × 10.0k was used to analyze the surface morphology of the electrode. The test was conducted at a bias voltage of 5 V and a current of 7 μA. A D8 Advance (Bruker, Germany) X-ray diffraction analyzer (XRD) was used to analyze the crystallization of the sample. Cu and Kα were used as the radiation sources. Microstructure characterization and elemental analysis were carried out using a Hitachi TM3000 benchtop scanning electron microscope (SEM) equipped with an X-ray energy dispersive spectrometer (EDS) detector. EDS point analysis and elemental plotting were carried out at an accelerated voltage of 15 kV to determine the distribution of constituent elements in the composite material.

### 2.4 Electrochemical measurements

Electrochemical tests were conducted using three-electrode tests. Linear voltammetry (LSV) and cyclic voltammetry (CV) tests were carried out using the CHI660 electrochemical workstation (Chenhua). The linear scanning voltammetry (LSV) test was carried out in a standard three-electrode system: a platinum sheet was used as the counter electrode, a saturated calomel electrode as the reference electrode, and the anode material to be tested as the working electrode. A solution of 0.1 M perchloric acid was selected as the electrolyte. The scanning rate was 50 mV s^−1^, and the measurement was carried out by adjusting the initial potential and the terminal potential. The electrochemical impedance test (EIS) was conducted using the ZAHNER electrochemical workstation. It was carried out in a 0.1 M HClO_4_ solution, with a bias voltage of 1.9 V and a sine wave with an amplitude of 10 mV applied, and the frequency range was 1–105 Hz.

### 2.5 Calculation and evaluation

First, 1 g of product was weighed and put into a 250 mL triangle cup. Then, 100 mL water was added until the sample was completely dissolved and the phenolphthalein indicator was subsequently introduced. This solution was titrated with a sodium hydroxide standard solution until it turned reddish, and stopped without fading for 30 seconds. The content of succinic acidic was then calculated by formula (1):^19^1
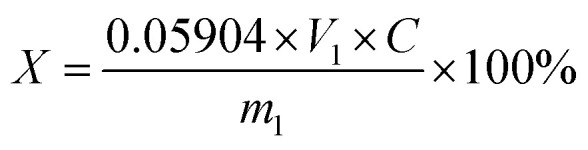
2
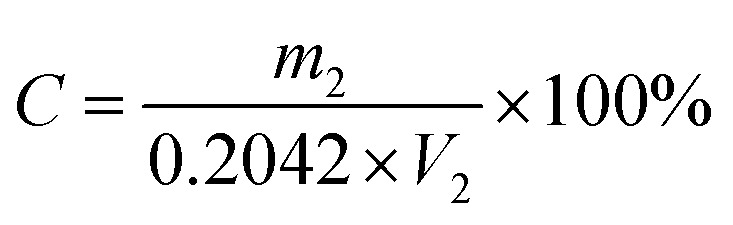
where *X* is the succinic acid content (%), *V*_1_ is the consumption volume of the NaOH standard solution (mL); *C* is the concentration of the NaOH standard solution (mol L^−1^); *m*_1_ is the mass of the succinic acid sample (g); 0.05904 refers to the mass of the succinic acid in grams equivalent to 1.00 mL NaOH standard solution (*C*_NaOH_ = 1.000 mol L^−1^); *V*_2_ is the volume of the NaOH solution (mL); *m*_2_ is the mass of potassium hydrogen phthalate (g); 0.2042 refers to the mass of potassium hydrogen phthalate in grams equivalent to *C*_NaOH_ = 1.000 mol L^−1^.

The ratio of the actual yield of the product to the theoretical yield^19^ was calculated by the following formula:3
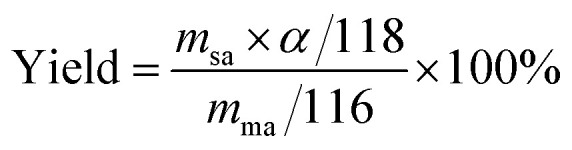


Current efficiency is an important technical and economic index in the process of electrosynthesis.^19^ Its calculation formula is as follows:4
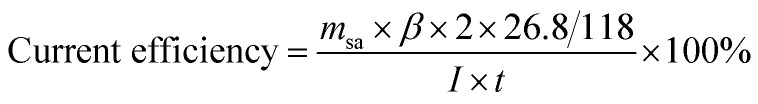
where, *m*_ma_ is the mass of maleic acid (g); *m*_sa_ is the mass of the succinic acid output (g); *I* is the applied current (A); *t* is electrolytic time; *α* is the purity of succinic acid; and *β* is the mass fraction of succinic acid in the electrolytic finishing solution.

## 3. Results and discussion

**Fig. 2:**
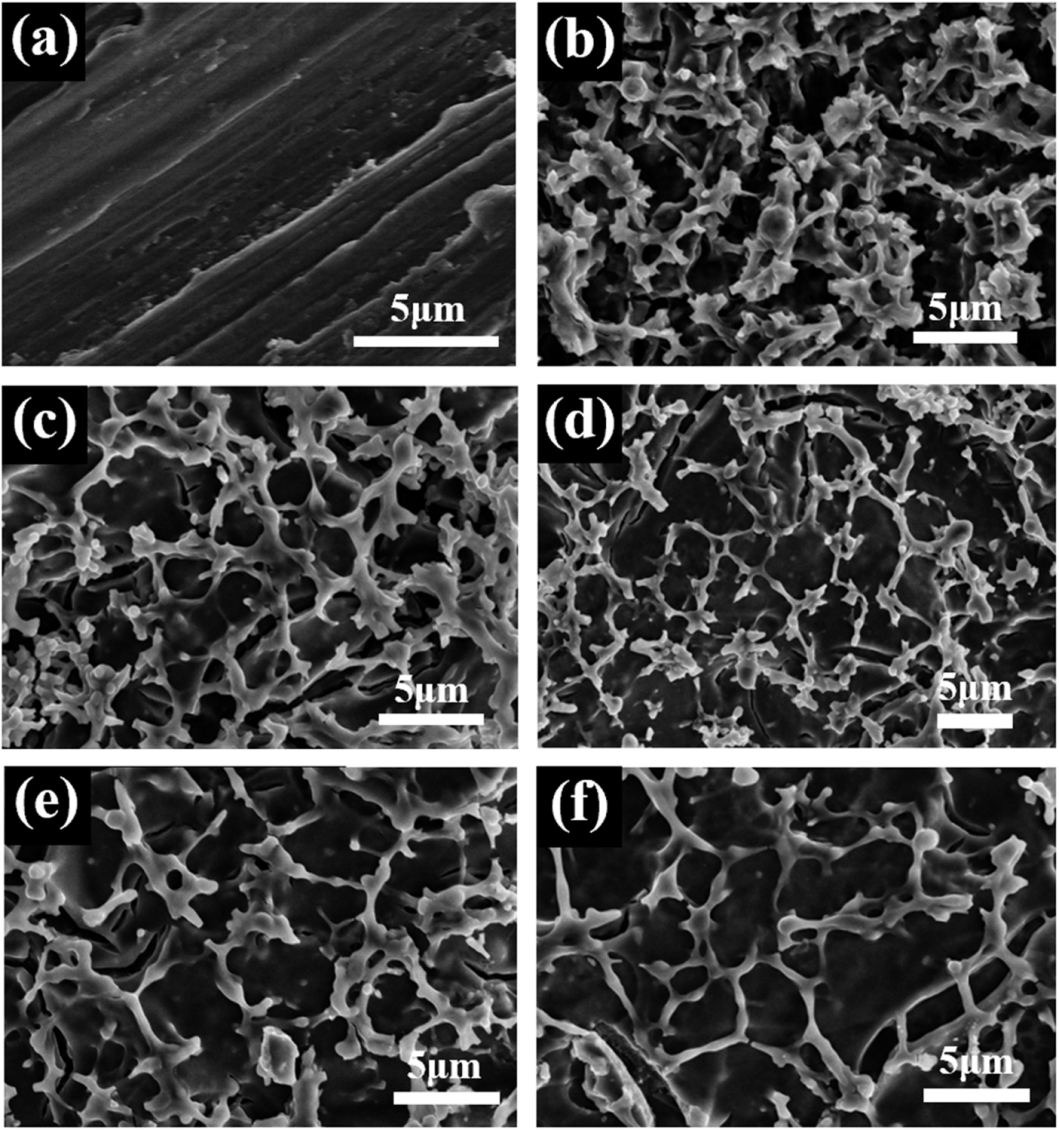
SEM images: (a) pure Ti, (b) 0-Y electrode, (c) 0.002-Y electrode, (d) 0.004-Y electrode, (e) 0.006-Y electrode, and (f) 0.008-Y electrode.

The morphologies of the Ti substrate and Ti/TiO_2_ film electrodes with different concentrations of Y element doping were observed by scanning electron microscopy (SEM). As can be seen from Fig. 2(a), the surface of pure Ti is smooth. This structure is not conducive to the transfer of electrons from the substrate to the TiO_2_ film. As can be seen from Fig. 2(b), the surface of the Ti/TiO_2_ electrode substrate presents a porous network structure. The root of the porous film is connected to the titanium substrate, and the longitudinal distribution of pores is interlaced, with serious corrosion between the connections and uneven pore size of ∼4 μm. After doping a small amount of Y element, the film layer becomes thinner, the morphology of pores becomes more obvious, and the pore size increases to a certain extent. The pore structure has a large specific surface area, which increases the catalytic active site on the electrode surface, and has a positive effect on improving the current efficiency of the electrode reduction of maleic acid to succinic acid, as shown in Fig. 2c–e. In addition, it can be seen that there are more cracks in the titanium substrate of the 0.002-Y and 0.004-Y electrodes, which is conducive to the infiltration of electrolytes into the titanium substrate, thus accelerating the passivation failure of the electrode. In comparison, the 0.006-Y electrode has fewer cracks, which is helpful in prolonging the service life of the electrode. Fig. 2(f) shows the surface changes after the doping ratio of the Y element is further increased. The porous network structure on the surface of the titanium matrix is significantly reduced, which is not conducive to the electrocatalytic reduction of maleic acid to succinic acid by the electrode.

**Fig. 3:**
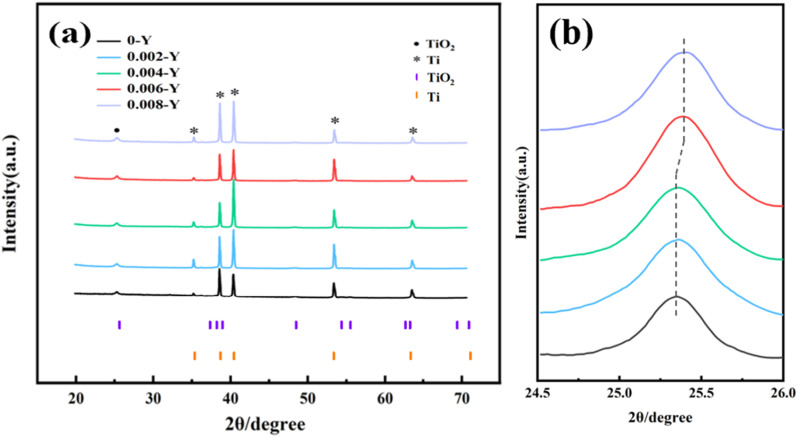
(a) XRD patterns of different electrodes and (b) partially enlarged XRD patterns.

We studied the effect of different Y doping ratios on the crystal quality of the titanium dioxide thin films. Fig. 3(a) shows the XRD patterns of TiO_2_-based materials with different Y doping amounts (0.02, 0.04, 0.06, 0.08). The main diffraction peaks of all samples were well matched with the standard cards of the anatase phases TiO_2_ and Ti. We can see that the purple color in the picture is marked as the TiO_2_ standard card and the orange color is marked as the Ti standard card. The diffraction peaks at 2*θ* values of 35.1°, 38.7°, 40.5°, 53.1°, and 63.2° correspond to the (100), (002), (101), (102), and (110) crystal planes of metal Ti, respectively.^20^ The strongest characteristic diffraction peaks of anatase-type TiO_2_ present in the sample at 2*θ* of 25.2° were attributed to the (101) crystal plane of the anatase phase.^21,22^ Unfortunately, other characteristic diffraction peaks of anatase-type TiO_2_ were not observed, which is possibly due to the limited thickness of the prepared film.^23^ In addition, there was no characteristic peak of Y oxide, indicating that TiO_2_ was the main component of the film layer on the electrode surface. This phenomenon may be caused by the fact that trace Y ions entered the TiO_2_ lattice interstitially or *via* displacement, instead of forming an ordered lattice structure. It is also possible that the doping content of element Y is too low, resulting in the inability to be detected by XRD. Subsequently, the crystal plane (110) was selected for careful observation, as shown in Fig. 3(b). By analyzing the diffraction peak intensity of plane (101), it can be seen that the diffraction peak intensity increases with increasing Y concentration. It then reaches the optimal value when the concentration is 0.006 M, while the film crystallinity decreases when the concentration is further increased to 0.008 M. At the same time, it can be seen that the increase of concentration has little effect on the location migration of the diffraction peak, which can thus be ignored. However, when the concentration increases to 0.006 M, the migration is more obvious.

**Fig. 4:**
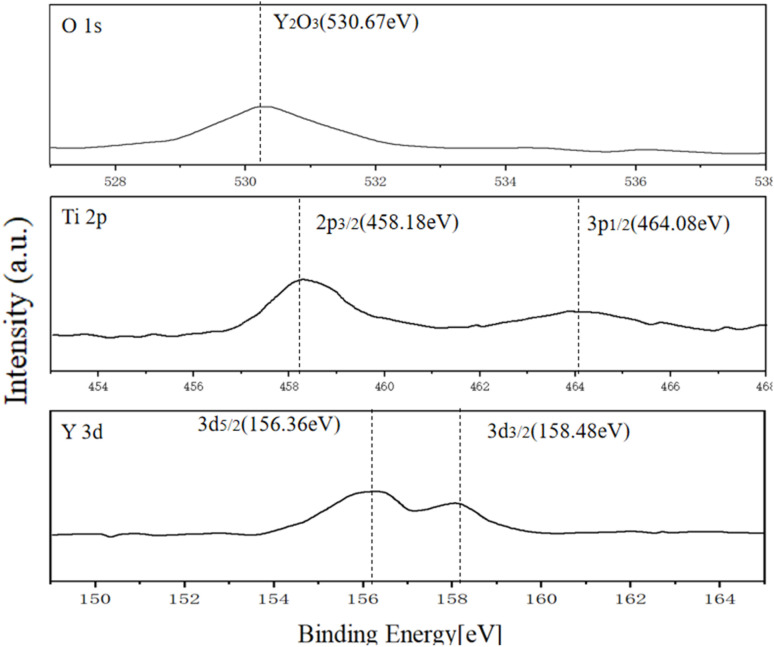
XPS results of O 1s, Ti 2p and Y 3d.

As shown in Fig. 4, the XPS spectra of Y-doped Ti/TiO_2_ composites reveal the chemical states of the elements on the material surface and their interactions. The Ti 2p spectrum shows double peaks at the binding energies of 458.18 eV and 464.08 eV, corresponding to the Ti 2p_3/2_ and Ti 2p_1/2_ orbitals, respectively, confirming the oxidation state of Ti^4+^. The spectral peak of O 1s is located at 530.67 eV. It is notable that the Y 3d spectrum exhibited double peaks at the binding energies of 156.36 eV and 158.48 eV, corresponding to the Y 3d_5/2_ and Y 3d_3/2_ orbitals, respectively, indicating that Y exists in the +3 oxidation state. In conclusion, the XPS results confirm that Y_2_O_3_ and TiO_2_ form a stable composite structure with uniform surface chemical states, providing favorable conditions for the electrocatalytic or interfacial reactivity of the material.

**Fig. 5:**
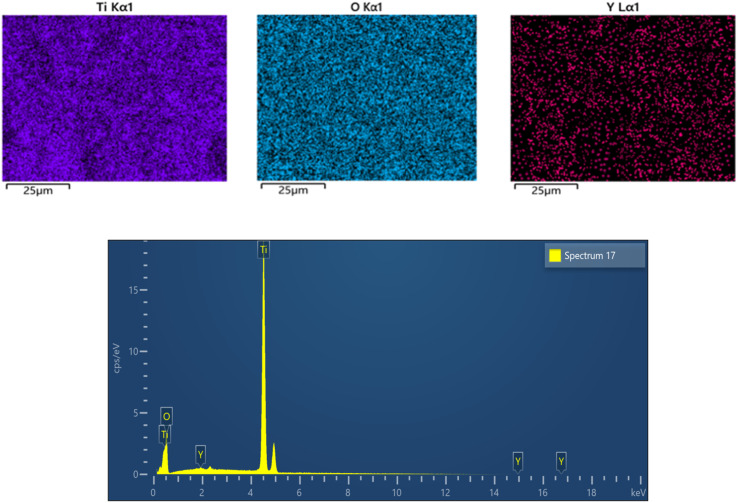
EDS of the 0.006-Y electrodes.

By performing the elemental scanning on the surface of the modified Y doped TiO_2_, it can be seen from Fig. 5 that the TiO_2_ film layer contains Ti, O and Y elements, and the Y ions are uniformly distributed in the film. This indicates that the Y element has been successfully introduced into the TiO_2_ film layer. The Ti element, as the dominant component of the matrix, presents a uniform distribution and mainly originates from the metallic Ti substrate or the TiO_2_ phase. The O element signal widely covers the detection area, and its content is close to the theoretical values of the oxidation states of TiO_2_ and Y_2_O_3_. However, due to the extremely low Y doping amount, the oxygen proportion is still mainly TiO_2_. It can also be seen from the following figure that the content of Ti is extremely high, while the Y element is not obvious in the figure due to its low doping content. From Table 1, we can see the content proportions of each element.

**Table 1:** Element distribution table for the 0.006-Y electrode

Element	Line	Weight%	wt% sigma	Atom%
O	K	35.38	0.45	62.17
Ti	K	64.29	0.45	37.73
Y	L	0.32	0.10	0.10

**Fig. 6:**
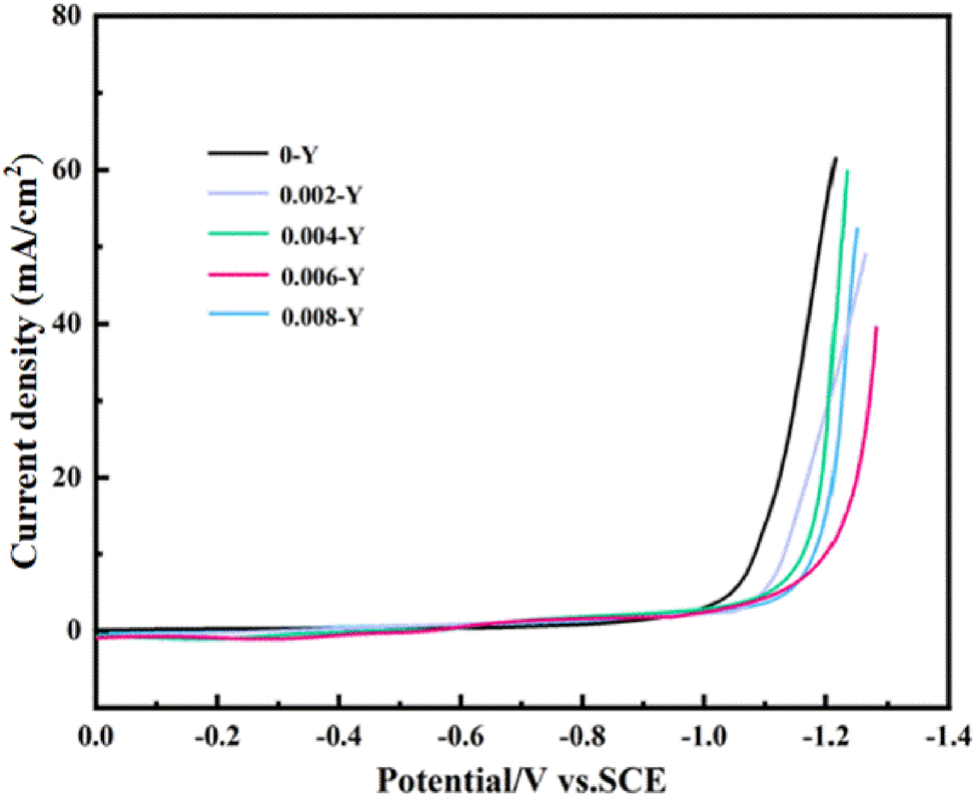
LSV curves of different electrodes at 1 M H_2_SO_4_.

The cathode provides a reaction site for the electrocatalytic reduction synthesis of organic compounds. Under the initial conditions, the main reaction usually cannot occur spontaneously. As the catalyst of the electrocatalytic reaction process, the cathode plays a crucial role. Cathodic reduction of organic matter is a complicated process, which is often accompanied by side reactions. The main side reaction is hydrogen evolution. A high hydrogen evolution potential indicates that it is difficult to precipitate hydrogen without affecting the progress of the catalytic reduction reaction. Therefore, materials with high hydrogen evolution potential should be selected for electrochemical reactions that are not aimed at achieving hydrogen evolution reduction. Fig. 6 shows the linear scanning voltammetry curves of the Ti/TiO_2_ electrodes with different Y-doping amounts in 1 M H_2_SO_4_ solution (scanning speed, 50 mV s^−1^). The cathode reaction process can be analyzed through the two stages of the hydrogen evolution polarization curve. The first stage is a relatively smooth curve, where the current density remains close to zero, and the structure of the electric layer on the cathode surface is changed. In the second stage, the curve jumps, the current density increases, and the hydrogen evolution rate of the electrode begins to increase. It can be seen from the figure that with the increase of the Y doping amount, the hydrogen evolution potentials corresponding to different electrodes are −1.03 V, −1.07 V, −1.17 V, −1.22 V, and −1.19 V. Hydrogen precipitates only when the hydrogen evolution overpotential of the 0.006-Y electrode drops to −1.22 V. It is difficult for the hydrogen evolution side reaction to affect the electrocatalytic reduction of maleic acid. This shows that the appropriately Y-doped Ti/TiO_2_ electrode is more beneficial to the electrocatalytic reduction of maleic acid to succinic acid than the pure Ti/TiO_2_ electrode.

**Fig. 7:**
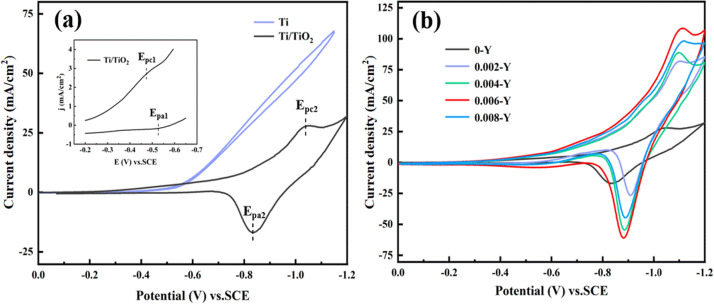
(a) CV curves of the Ti and Ti/TiO_2_ electrodes at 1 M H_2_SO_4_. (b) CV curves of different electrodes at 1 M H_2_SO_4_.

Fig. 7(a) shows the CV curves of pure Ti and Ti/TiO_2_ electrodes at 1 M H_2_SO_4_. In the potential range of 0 to −1.2 V, the pure Ti electrode does not show redox peaks, while the Ti/TiO_2_ electrode has two pairs of redox peaks. The corresponding redox peaks of TiO_2_/Ti_2_O_3_ and TiO_2_/Ti(OH)_3_ are about −0.48 V and −1.04 V, respectively. The oxidation peak potentials *E*_pa_1__ and *E*_pa_2__ are about −0.54 V and −0.83 V, respectively. The TiO_2_/Ti_2_O_3_ redox peak potential difference (Δ*E*_p_1__) is about 60 mV, and the positive and negative peak current |*i*_pa_|/|*i*_pc_| ratio is about 1. The results are independent of the scanning speed, and the reaction is reversible. The TiO_2_/Ti (OH)_3_ redox peak potential difference (Δ*E*_p_1__) is about 210 mV, and the positive and negative peak current |*i*_pa_|/|*i*_pc_| ratio is greater than 1. The asymmetric cycle curve exhibited upward and downward trends, indicating that the reaction is reversible. The reaction formula corresponding to the two pairs of redox peaks of the Ti/TiO_2_ film electrode is as follows:^24^

5 TiO_2_ + 2H^+^ + 2e^−^ ↔ Ti_2_O_3_ + H_2_O

6 TiO_2_ + H_2_O + H^+^ + e^−^ ↔ Ti(OH)_3_

Cyclic voltammetry was used to study the electrocatalytic activity of the Y-doped nano-TiO_2_ thin film electrodes with different Y/Ti molar ratios in 1 M H_2_SO_4_ solution, as shown in Fig. 7(b). It can be seen that with increasing Y ion concentration, the cathode peak current first increases and then decreases. When the Y/Ti molar ratio is 0.006, the peak cathode current is the largest. This peak of the cathode current is four times that of the undoped electrode. Thus, a small number of Y ions will indeed lead to an increase in the reduction peak current. However, an excess of Y ions will hinder the transfer of electrons, resulting in a decrease in the reduction peak current. In addition, the Y ion distorts the titanium dioxide lattice, resulting in a negative shift of the peak potential.

**Fig. 8:**
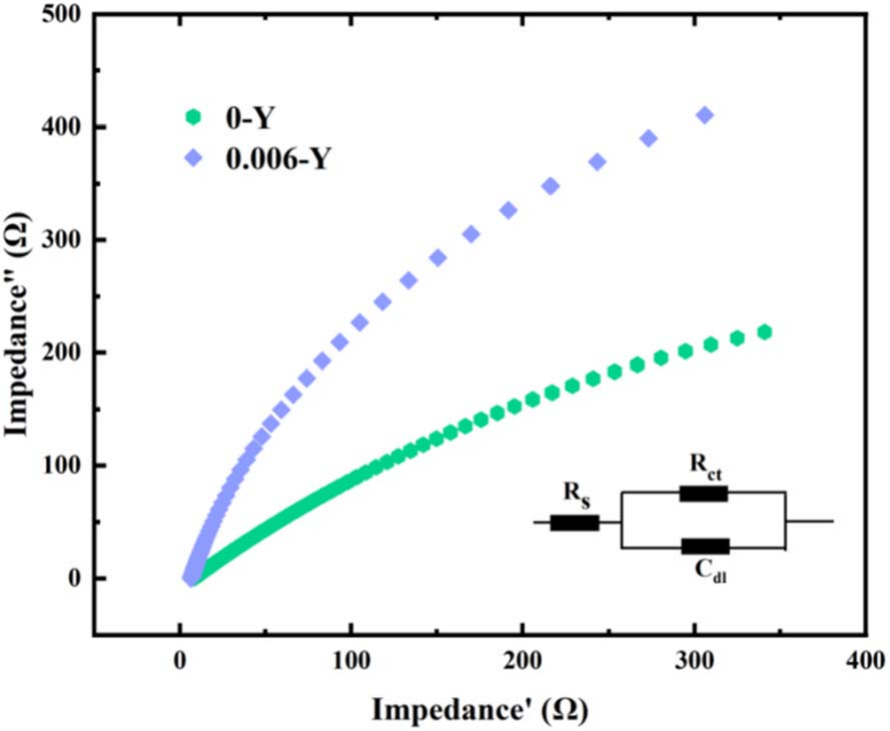
EIS curves of the 0-Y and 0.006-Y electrodes.

The interface impedance of the electrode can be characterized by the EIS test. The presence of impedance between interfaces will affect the current transmission of electrodes. The EIS measurement is an important tool for electrocatalytic performance analysis that reflects the charge transfer impedance in the catalytic process. In Fig. 8, compared with the unmodified electrode, the 0.006-Y electrode shows a lower charge-transfer resistance and a smaller semicircle region, which indicates that the electrode has a larger capacitance. The simulation results match the CV results. This may be due to the porous membrane structure of the electrode and the improved conductivity of ion introduction, which effectively reduces the charge transport.^25,26^ In addition, many active sites are exposed and ions are transported to the interface between the electrode and electrolyte. Therefore, the optimized electrode may greatly reduce the high energy consumption problem in practical applications.

**Fig. 9:**
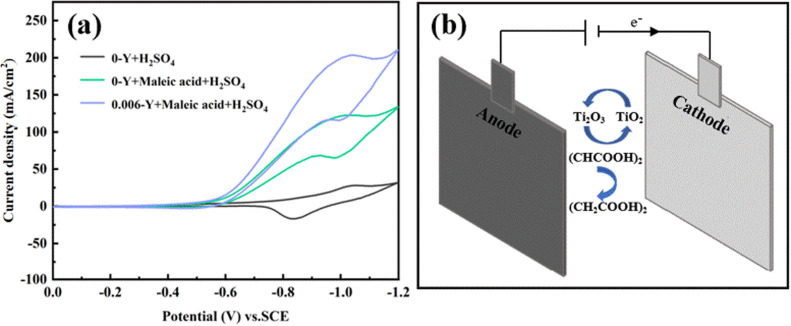
(a) CV curve of the 0-Y electrode in 1 M H_2_SO_4_ and 1 M maleic acid + 0.5 M H_2_SO_4_ solution and the CV curve of the 0.006-Y electrode in 1 M maleic acid + 0.5 M H_2_SO_4_ solution. (b) Indirect reduction of maleic acid to succinic acid on a Ti/TiO_2_ film electrode.

The CV curve of the 0-Y electrode in 1 M H_2_SO_4_, 1 M maleic acid + 0.5 M H_2_SO_4_ solution, and the CV curve of the 0.006-Y electrode in 1 M maleic acid + 0.5 M H_2_SO_4_ solution are shown in Fig. 9(a). In the solution containing maleic acid, there is an obvious reduction peak between −0.55 V and −1.1 V at the 0-Y electrode. The initial potential of the maleic acid reduction peak is −0.58 V, while that of the Ti^4+^ reduction peak is −0.73 V in H_2_SO_4_ solution. This indicates that the cathode can reduce maleic acid at a lower potential and has high catalytic activity. There is an upward adsorption peak in the back sweep process, but no oxidation peak corresponding to the reduction peak, indicating that the reduction of maleic acid is irreversible. By comparison, the peak current of the maleic acid reduction of the 0.006-Y electrode is about twice that of the undoped electrode, which confirms again that the doping of the Y ion can improve the catalytic activity of the electrode.

In addition, the reduction potential of maleic acid is similar to the reversible potential of the TiO_2_/Ti_2_O_3_ electrode pair. Furthermore, the reduction potential of maleic acid is similar to the reversible potential of the TiO_2_/Ti_2_O_3_ redox couple. This similarity enables the Ti^4+^/Ti^3+^ species (TiO_2_/Ti_2_O_3_) on the Ti/TiO_2_ cathode surface to act as an electron transfer mediator, facilitating the reduction of maleic acid to succinic acid, as shown in Fig. 9(b). The electrocatalytic reduction produces a rapid catalytic reaction between Ti^3+^ and maleic acid on the electrode surface, and a large amount of Ti^4+^ is regenerated by chemical catalysis without electrode reaction. Thus, the oxidation peak disappears and maleic acid is indirectly reduced to succinic acid. This process is repeated continuously. The Ti/TiO_2_ cathode achieves heterogeneous electrocatalytic reduction of maleic acid.^27,28^ The equation is as follows:

7 2Ti^4+^ + e^−^ ↔ 2Ti^3+^

8 2Ti^3+^ + (CHCOOH)_2_ + 2H^+^ → 2Ti^4+^ + (CH_2_COOH)_2_

**Fig. 10:**
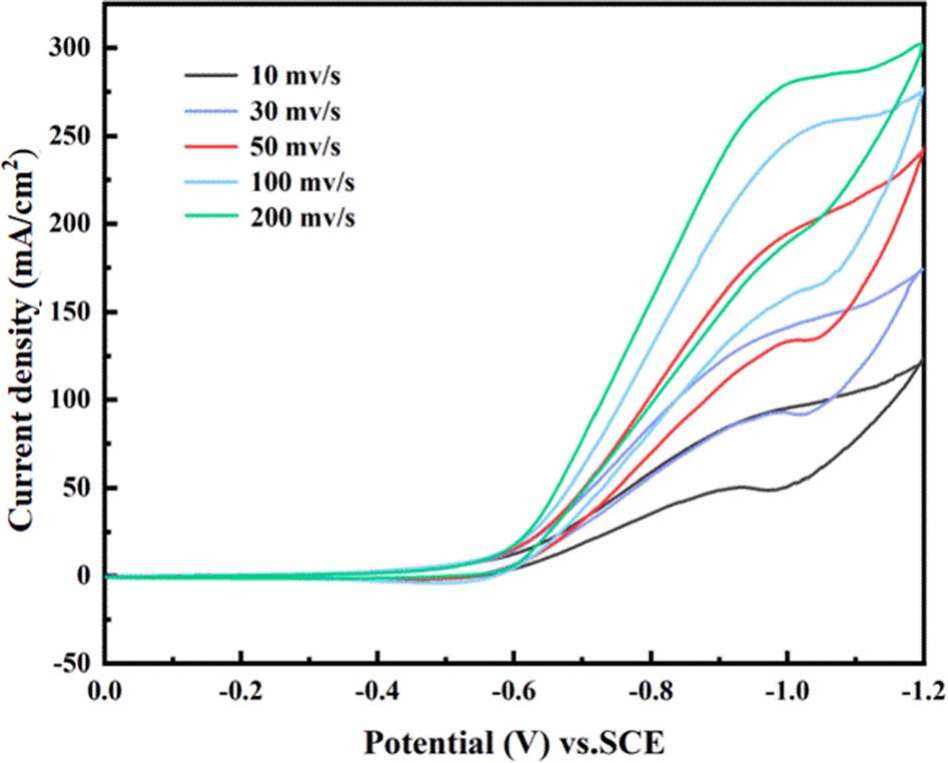
CV curves of maleic acid at different sweep speeds at 0.006-Y electrodes.

To study the relationship between different sweep rates and the reduction peak current, CV curves of the 0.006-Y electrode were tested in 1 M maleic acid and 0.5 M H_2_SO_4_ solution at different sweep rates. It can be concluded from Fig. 10 that there is a positive correlation between the scanning speed and the peak reduction current, which indicates that the reduction reaction of maleic acid on the TiO_2_ film electrode is very rapid. The scanning speed refers to the time required to reach the peak potential. A higher scanning speed results in a shorter time required to reach the peak potential. Correspondingly, a higher diffusion rate and faster charge transfer occur on the electrode surface, thus increasing the peak current. In addition, the potential corresponding to the reduction peak current migrates to the left to a certain extent with the increase in scanning speed, which further indicates that the electrocatalytic reduction of maleic acid to succinic acid is irreversible.

**Fig. 11:**
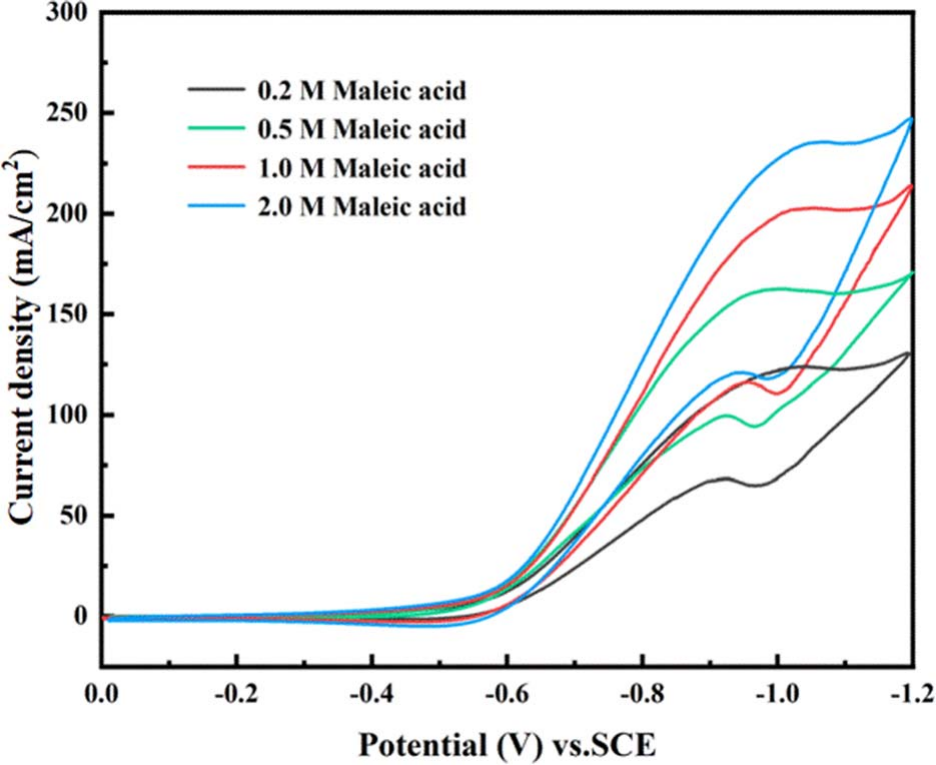
CV curves of maleic acid at different initial concentrations at the 0.006-Y electrode.

The influence of maleic acid concentration on the peak reduction current is shown in Fig. 11. It can be observed that the higher maleic acid concentration at the potential of −0.55 V to −1.1 V promotes the peak reduction current. In a high concentration of the maleic acid solution, a large number of maleic acid ions react with Ti^3+^ on the surface of the TiO_2_ film electrode, thus speeding up the electrocatalytic reduction reaction and increasing the reduction peak current. However, excessive maleic acid will delay the reaction time, which is not conducive to achieving higher current efficiency.

**Fig. 12:**
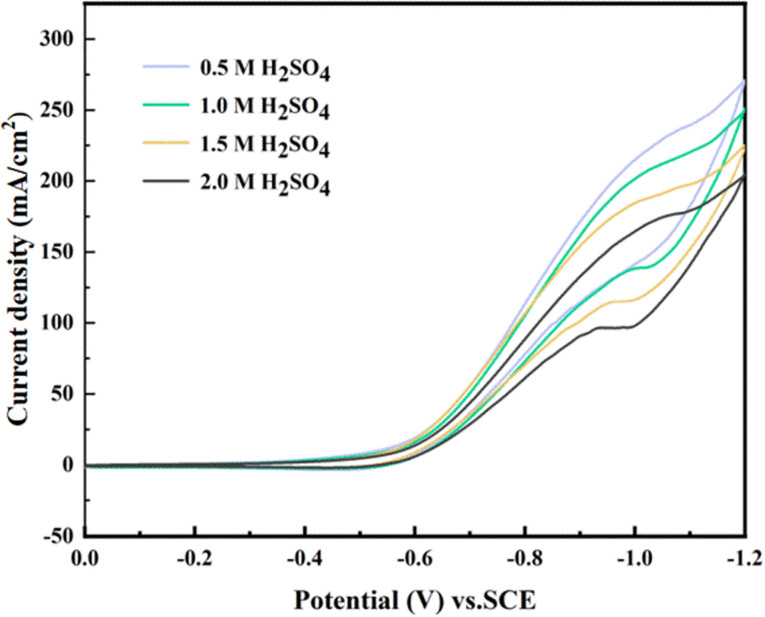
CV curves of maleic acid at the 0.006-Y electrode with different electrolyte concentrations.

Subsequently, we studied the influence of different H_2_SO_4_ concentrations on the reduction peak current, as shown in Fig. 12. The electrolyte concentration has little effect on the electrocatalytic reduction reaction, and the lower electrolyte concentration is conducive to the reduction of maleic acid. At a higher electrolyte concentration, the solution viscosity increases, thus increasing the product-desorbed diffusion resistance on the electrode surface. At the same time, the maleic acid replenishment rate to the electrode interface slows down. This results in a decreased maleic acid concentration in the electrode reaction layer, and the main reaction is inhibited. Therefore, more H^+^ is involved in the discharge reaction, which means that the cathode current efficiency is significantly reduced.^29^

**Fig. 13:**
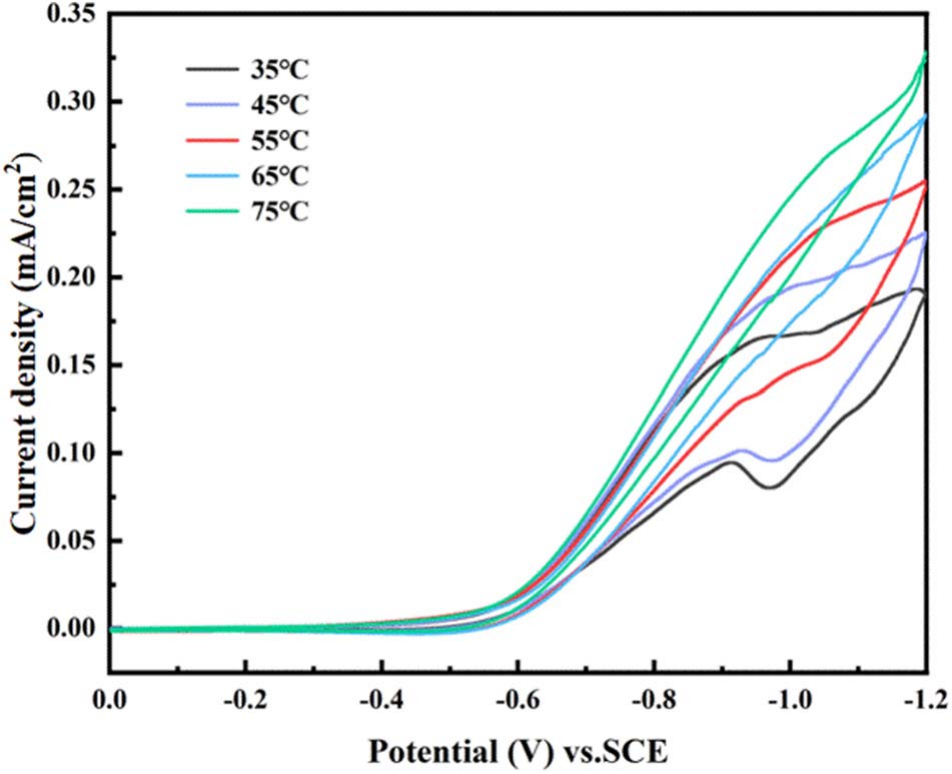
CV curves of maleic acid at the 0.006-Y electrode at different temperatures.

Finally, we discuss the influence of temperature change on the cathode reduction peak current. CV curves of the 0.006-Y electrode at different temperatures were tested in 1 M maleic acid and 0.5 M H_2_SO_4_ solution, as shown in Fig. 13. Low temperature is not conducive to improving the reduction peak current. This is because the solubility of maleic acid (780 g L^−1^) is much greater than that of succinic acid (80 g L^−1^) at room temperature. Furthermore, maleic acid is reduced on the cathode and succinic acid is precipitated in crystal form. The product is attached to the electrode surface, hindering the reaction between the active site on the electrode surface and maleic acid. It can be seen that when the temperature exceeds 65 °C, the cathode wave does not show a peak shape. At an excessively high temperature, the viscosity of the electrolyte is reduced, thus increasing the diffusion rate of the reactant. However, the excessively high temperature affects the increase in the tank pressure, resulting in the loss of energy consumption.^29,30^

The succinic acid products were separated by the difference in solubility between maleic acid and succinic acid. At the end of the reaction, the electrolytic liquid was cooled, crystallized, purified, pumped, and dried to obtain the product for weighing. It was found that the yield reached 91%, the current efficiency reached 96.3%, and the purity of succinic acid is more than 99%. Compared with the 0-Y electrode, the current efficiency is improved by 14%. As shown in Table 2, we can see the advantages demonstrated by the electrode in this paper when compared with other electrodes.

**Table 2:** Comparison of our work with other similar studies

Electrode reported in this work	Electrode used in previous work	Improvement of materials
Anodes: Ti/TiO_2_–Y_2_O_3_	Anodes: Ti/nano-TiO_2_	The yield of the Ti/TiO_2_–Y_2_O_3_ electrode reached 91% for the Ti/nano-TiO_2_ electrode. The current efficiency of the Ti/TiO_2_–Y_2_O_3_ electrode is also higher than that of the Ti/nano-TiO_2_ electrode, reaching 96.3%
Cathodes: stainless steel plate	Cathodes: stainless steel plate	
Anodes: Ti/TiO_2_–Y_2_O_3_	Anodes: Ti/nanoTiO_2_-Pt	The current efficiency of the Ti/TiO_2_–Y_2_O_3_ single anode (96.3%) is higher than that of the Ti/nanoTiO_2_–Pt anode (84%). Economically speaking, the price of Y is much lower than that of Pt, which is of more practical significance for the industrial synthesis of succinic acid
Cathodes: stainless steel plate	Cathodes: Ti/nanoTiO_2_	
Anodes: Ti/TiO_2_–Y_2_O_3_	Anodes: Pb	The doping content of Y in the Ti/TiO_2_–Y_2_O_3_ electrode is extremely low. The toxicity of Pb is much higher than that of Y. Therefore, from environmental protection and safety perspectives, Ti/TiO_2_–Y_2_O_3_ is superior to Pb electrodes
Cathodes: stainless steel plate	Cathodes: Ti	

**Fig. 14:**
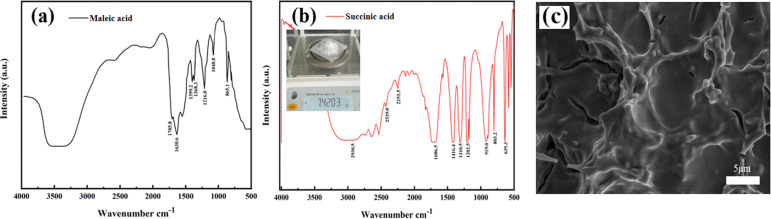
(a) FT-IR spectrum of maleic acid. (b) FT-IR spectrum of succinic acid products. (c) SEM image of the electrode after continuous electrolysis for 24 hours.

The melting point of the obtained product was determined to be 184–188 °C, which is consistent with the literature value of succinic acid (185–187 °C). The product was further characterized by FT-IR, which is shown in Fig. 14(b). The weaker absorption peak at 2930.5 cm^−1^ is attributed to the C–H stretching vibration peak, and the wider peak at 1688.5 cm^−1^ corresponds to the C=O stretching vibration absorption. The strong absorption at 1416.4 cm^−1^ indicates the presence of –COOH. The stretching vibration frequency range of the C=C bond is 1680–1620 cm^−1^, and there is no absorption of the C=C bond in the range of 1680–1620 cm^−1^, indicating the purity of the succinic acid product. By comparison with Fig. 14(a), it can be seen that the initial raw material of maleic acid has a strong absorption at 1630.6 cm^−1^, which is attributed to C=C. Upon reduction into succinic acid through the cathode reduction reaction, this double bond disappears. According to the above analysis, it can be confirmed that the prepared product is succinic acid.

Fig. 14(b) shows the actual weighing process of the prepared sample of succinic acid (C_4_H_6_O_4_). As shown in the figure, the white crystalline succinic acid powder is placed in the weighing boat of the analytical balance. The loose and uniform particle morphology indicates that the sample has a high degree of crystallinity after purification. To reduce interference from environmental humidity, the weighing operation was completed under a dry atmosphere. This photograph visually reflects the physical state of the sample and the control of weighing accuracy. As shown in Fig. 14(c), we carried out the SEM characterization after electrolysis. The results show that after continuous electrolysis for 24 hours, the macroporous TiO_2_ layer remains intact without delamination or cracking, indicating the good structural stability of the electrode.

**Fig. 15:**
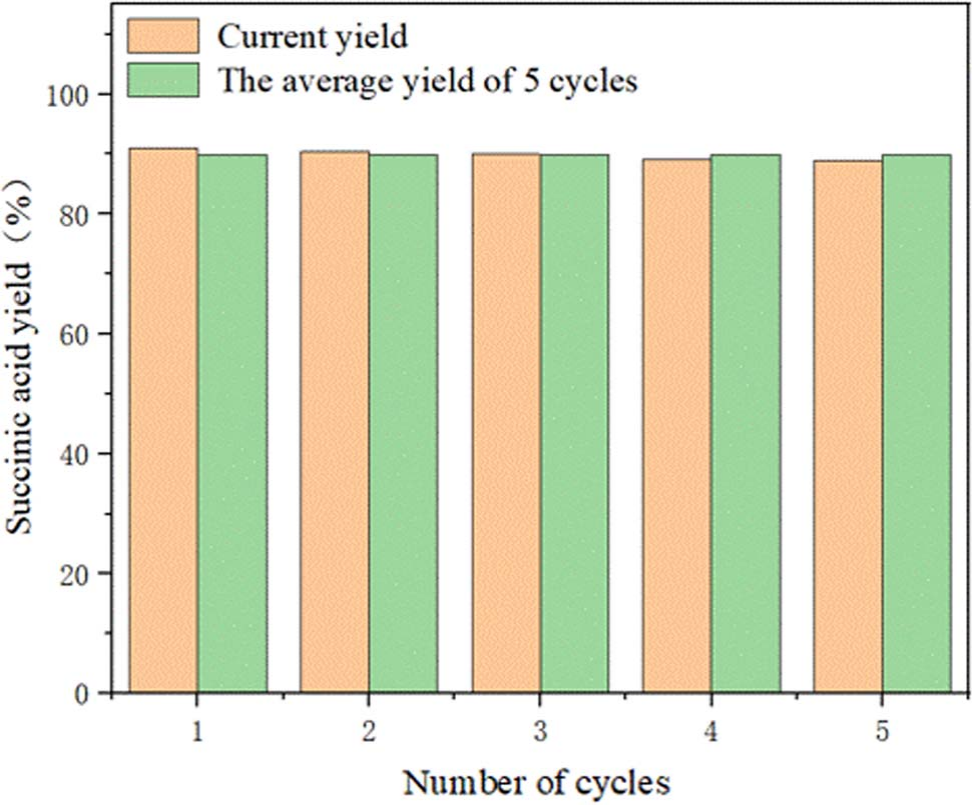
. The yield graph of succinic acid after five cycles of tests on the same electrode.

From Fig. 15, we can see that the average value after 5 cycles is very different from that of the fourth cycle. At the same time, it can also be seen that after 5 cycles, the yield of succinic acid is still close to 90%, which indicates that this electrode has good repeatability.

## 4. Conclusion

In this paper, the sol–gel method was used to fabricate a Y-doped Ti/TiO_2_ film electrode. SEM characterization found that the macroporous morphology of the film became more obvious upon the addition of Y ions, and the substrate cracks were also improved. LSV and CV confirmed that the hydrogen evolution potential of the Y-doped Ti/TiO_2_ membrane electrode was increased. At the optimal Y/Ti molar ratio of 0.006, the hydrogen evolution potential reached 1.22 V and the hydrogen evolution side reaction was effectively inhibited. The reduction peak current density in the maleic acid solution is as high as 0.21 A cm^−2^, which is 1.7 times that of the undoped electrode, indicating that the addition of trace Y element improves the electrocatalytic reduction performance of the electrode. Considering that the cathode can reduce maleic acid at a lower potential and has higher catalytic activity, the cathode potential is controlled between −0.6 V and −1.2 V for the electrosynthesis of succinic acid. When the optimized reaction temperature is 50 °C, the electrosynthesis yield of succinic acid reaches 91% and the current efficiency is 96.3%. However, some limitations of this approach include the relatively long preparation cycle of this electrode and the scalability of rare earth doping, which remain to be addressed. Future research should address the following: (1) shorten the electrode preparation cycle, (2) optimize the economy, and (3) design for sustainability by embedding rare earth recovery units.

## Author contributions

All the authors contributed to study conception and design. Material preparation as well as data collection and analysis were performed by Shaojie Hong, Fanhua Yu, Bin Guo, Xiangqian Ren and Xingfu Zhou. The first draft of the manuscript was written by Shaojie Hong, and all the authors commented on the previous versions of the manuscript. All the authors read and approved the final manuscript.

## Conflicts of interest

On behalf of all the authors, the corresponding author declares that the authors have no financial interests or personal relationships with other people or organizations that could have appeared to inappropriately influence the work reported in this paper.

## Data Availability

Data will be made available upon request.
